# 18F-FDG PET/CT-Informed Multidisciplinary Reassessment Modifies Surgical Strategy in Patients with Metastatic Bone Disease: Exploratory Predictors of Decision Change in an Orthopedic Oncology Board

**DOI:** 10.3390/jcm15166436

**Published:** 2026-08-20

**Authors:** Erkan Akgun, H. Emre Tepedelenlioğlu, Serkan Aydin, Turgut Yurdakul, S. Sinan Gültekin, H. Bilgehan Çevik

**Affiliations:** 1 Department of Orthopedics and Traumatology, Ankara Etlik City Hospital, Halil Sezai Korkmaz St., Varlık, Ankara 06170, Türkiye; hemretepe@hotmail.com (H.E.T.); drsaydin2003@yahoo.com (S.A.); drturgutyurdakul@gmail.com (T.Y.); bilgehancevik@gmail.com (H.B.Ç.); 2 Department of Nuclear Medicine, Ankara Etlik City Hospital, Halil Sezai Korkmaz St., Varlık, Ankara 06170, Türkiye; gultekinsinan@gmail.com

**Keywords:** bone metastases, 18F-FDG PET/CT, surgical decision-making, multidisciplinary tumor board, orthopedic oncology, SUVmax

## Abstract

**Background/Objectives**: Bone is the third most common site of solid-tumor metastasis, and surgical management spans a wide spectrum from observation to curative en bloc resection in selected patients. 18F-FDG PET/CT has been reported to alter oncologic management in 20% to 45% of patients across diverse settings, yet its specific impact on surgical decision-making within a multidisciplinary orthopedic oncology board (MOOB) reassessment and the predictors of decision change remain undefined. **Methods**: We retrospectively analyzed consecutive patients with histopathologically confirmed bone metastases evaluated at a tertiary-care MOOB between February 2023 and February 2026. For each patient, the surgical plan was recorded twice: first by the orthopedic team based on available conventional imaging (radiography, computed tomography, magnetic resonance imaging) and then as the final PET/CT-informed MOOB review. This design evaluates changes in decision-making intent after PET/CT-informed multidisciplinary reassessment, rather than the isolated causal effect of PET/CT or actual surgical implementation. Surgical plans were categorized as no surgery, palliative stabilization, palliative resection, or curative-intent resection, and changes were classified as no change, escalation, de-escalation, or cancellation. **Results**: Among 73 patients (mean age 64.7 ± 12.1 years; 53.4% male), the most frequent primaries were lung (37.0%), breast (21.9%), and renal cell carcinoma (13.7%). Following PET/CT-informed MOOB reassessment, the intended surgical plan changed in 53.4% of cases (*p* < 0.001), comprising surgery cancellation (27.4%), escalation (17.8%), and de-escalation (8.2%). Patients directed to no surgery increased fourfold (6.8% to 28.8%), and curative resection emerged as a post-reassessment recommendation (0% to 10.9%). SUVmax did not predict overall decision change, but higher SUVmax was associated with cancellation/de-escalation in the directional analysis. In exploratory multivariable analysis, polymetastatic disease (OR 0.051; 95% CI 0.005 to 0.547; *p* = 0.014), long-bone diaphyseal location (OR 0.133; *p* = 0.006), and higher Eastern Cooperative Oncology Group (ECOG) performance score (OR 2.109 per point; *p* = 0.018) independently predicted decision change. **Conclusions**: PET/CT-informed MOOB reassessment was associated with substantial changes in intended surgical strategy for metastatic bone disease. The findings support careful multidisciplinary integration of metabolic imaging, metastatic burden, anatomic location, and performance status, but should be regarded as hypothesis-generating until validated prospectively with data on treatment delivery and downstream outcomes.

## 1. Introduction

The skeleton is the third most common site of metastatic involvement from solid tumors, following the lung and liver, and skeletal metastases substantially compromise patients’ functional capacity and quality of life [1]. Over the past decade, advances in systemic cancer therapy have prolonged overall survival, and a growing proportion of these patients now present to orthopedic clinics with established bone metastases. Therapeutic goals include pain control, prevention of pathological fracture, durable local control, and, in carefully selected cases, cure through wide resection [2,3]. Achieving these goals requires integrating multiple patient- and disease-related variables, including overall health status, metastatic burden, primary tumor biology, and response to systemic therapy. Because these factors interact in complex, often competing ways, treatment decisions are best made within a multidisciplinary orthopedic oncology board (MOOB) that integrates expertise in orthopedic oncology, medical oncology, radiation oncology, radiology, and pathology [4].

18F-fluorodeoxyglucose positron emission tomography/computed tomography (18F-FDG PET/CT) has become a cornerstone of modern oncologic imaging and is widely used for staging, response assessment, and surveillance of recurrence [5,6,7]. Computed tomography (CT) and whole-body bone scintigraphy remain commonly used for skeletal staging, but both have well-recognized limitations and may underestimate the true extent of systemic disease [8,9]. By providing the maximum standardized uptake value (SUVmax), 18F-FDG PET/CT offers a quantitative measure of lesion metabolic activity that informs assessment of tumor biology and prognosis [5,10]. Nevertheless, the specific contribution of 18F-FDG PET/CT to surgical decision-making is difficult to isolate when imaging is reviewed within a MOOB, because the final recommendation also incorporates medical oncology, radiation oncology, radiology, and performance-status information. Although a limited number of studies have reported that 18F-FDG PET/CT alters overall treatment strategy in approximately 20% to 45% of selected oncologic populations [7,11], the magnitude of this effect in contemporary orthopedic oncology practice and the independent predictors of decision change remain undefined.

Beyond its diagnostic role, 18F-FDG PET/CT has been shown to influence treatment planning across various ontological settings and to support the personalization of management strategies [12,13]. In metastatic bone disease, the systematic incorporation of PET/CT findings, along with SUVmax, metastatic burden, and anatomic location and patient performance status may collectively refine both the indication for and the extent of surgical intervention. However, SUVmax is affected by tumor histology, lesion size, lytic or blastic morphology, prior therapy, scanner/protocol factors, and glycemic status; therefore, it should be interpreted as one component of multidisciplinary decision-making rather than as a universal marker of tumor aggressiveness.

The primary aim of this study was to quantify changes in intended surgical treatment plans after PET/CT-informed MOOB reassessment in patients with bone metastases. The secondary aim was to identify the independent clinical, anatomic, and metabolic predictors of decision change to support more targeted PET/CT use and individualized treatment planning in multidisciplinary orthopedic oncology practice.

## 2. Materials and Methods

This single-center retrospective cohort study was conducted at Ankara Etlik City Hospital, a tertiary referral center. The study was approved by the local Institutional Review Board (AESH-BADEK1-2026-203, 25 March 2026) and was conducted in accordance with the principles of the Declaration of Helsinki. Given the retrospective design and the use of de-identified data, the requirement for written informed consent was waived. The institutional electronic medical record system was searched for consecutive patients evaluated at the orthopedic oncology clinic between 1 February 2023 and 6 February 2026. All bone metastases were histopathologically confirmed, and every included patient was discussed at the MOOB. No formal sample size calculation was performed; all consecutive patients meeting eligibility criteria during the study period were included. Patients with missing key variables were excluded a priori in accordance with the exclusion criteria below, and no imputation was performed. Because the excluded screening records did not consistently retain the same clinical, imaging, and MOOB variables required for analysis, a reliable comparison between included and excluded patients could not be performed.

Eligible patients were adults (≥18 years) with histopathologically confirmed bone metastasis from a primary malignancy originating in an organ other than bone or hematopoietic tissue, who had been evaluated both at the initial departmental orthopedic team meeting and subsequently at the MOOB, with 18F-FDG PET/CT available at the time of MOOB review. Patients were excluded if they had insufficient clinical or pathological data.

Recorded variables included demographic data (age, gender), primary tumor type, and Eastern Cooperative Oncology Group (ECOG) performance status [14]. Estimated survival was documented at the time of MOOB review by the treating medical oncology team, based on disease extent, response to systemic therapy, and patient comorbidities, and was abstracted as a contextual variable for decision-making rather than used as a quantitative predictor in regression analysis. The index lesion was designated at the initial orthopedic team meeting, before PET/CT review, according to the dominant clinical or conventional imaging burden. The SUVmax used for analysis was then extracted from the official nuclear medicine report for this predesignated index lesion. The anatomic location of the index lesion was classified into three zones: Zone 1: axial skeleton and shoulder or pelvic girdles (spine, pelvis, sacrum, scapula, clavicle); Zone 2: long-bone epiphyses; Zone 3: long-bone diaphyses.

Overall metastatic burden was categorized as solitary (single lesion), oligometastatic (2 to 3 lesions), or polymetastatic (>3 lesions).

Skeletal involvement was assessed with plain radiographs, CT, magnetic resonance imaging (MRI) with contrast, and 18F-FDG PET/CT. Conventional imaging before the initial orthopedic plan was not mandated by a uniform protocol for every patient; rather, it reflected real-world decision-making and the imaging available at referral. All PET/CT examinations were performed on an integrated PET/CT scanner in accordance with the European Association of Nuclear Medicine procedure guidelines for tumor imaging, version 2.0 [15]. Patients fasted for at least 6 h prior to examination, and pre-injection capillary blood glucose levels were confirmed to be below 200 mg/dL. A weight-adjusted activity of 18F-FDG (approximately 3.5 to 4.0 MBq/kg) was administered intravenously, followed by a 60 ± 10 min uptake interval in a quiet, dimly lit room. Whole-body acquisition was performed from the skull base to the mid-thigh with the patient in the supine position and arms elevated above the head; in patients with suspected appendicular involvement below the mid-thigh, the field of view was extended to include the entire lower extremity. Low-dose CT was acquired first for attenuation correction and anatomic localization (120 kVp, automated tube current modulation, slice thickness 3 mm), followed by PET acquisition at 2 to 3 min per bed position. PET images were reconstructed using an ordered-subset expectation maximization (OSEM) iterative algorithm with CT-based attenuation correction and a Gaussian post-reconstruction filter. The SUVmax of the index lesion was extracted from the official nuclear medicine report and used for all analyses.

The surgical management plan was recorded at two prespecified time points: the initial departmental orthopedic team meeting, based on conventional imaging immediately after histopathologic confirmation of bone metastasis and before PET/CT review, and the final PET/CT-informed recommendation reached at the MOOB. At each time point, the surgical plan was classified into one of four categories: no surgery, palliative stabilization (e.g., internal fixation, intramedullary nailing, or cementoplasty without tumor removal), palliative resection defined as lesion resection or debulking intended primarily for symptom control, local mechanical control, or complication prevention without an expectation of complete systemic disease clearance, or curative resection (wide en bloc resection with the intent of complete tumor clearance). Changes between the two time points were classified into one of four categories: (1) no change; (2) escalation, defined as broader anatomic extent and/or greater surgical aggressiveness; (3) de-escalation, defined as narrower extent and/or reduced aggressiveness; and (4) cancellation, defined as withdrawal of any planned surgical intervention. For each patient whose plan was changed, the primary reason recorded in the MOOB minutes was abstracted and categorized as radiotherapy redirection, estimated survival, curative targeting, ECOG status, or detection of a new lesion. When multiple contributing factors were documented, the reason designated as primary in the MOOB minutes was used for analysis. Actual delivered treatment, margin status, post-cancellation fracture events, pain outcomes, local progression, survival, and functional outcomes were not systematically captured in the de-identified dataset.

### Statistical Analysis

All analyses were performed in R version 4.1.1 (R Foundation for Statistical Computing, Vienna, Austria). The Shapiro–Wilk test was used to assess the normality of continuous variables. Continuous data are reported as mean ± standard deviation (SD) or median (interquartile range [IQR]), as appropriate, and categorical data as number (percentage). Between-group comparisons of continuous variables used the Mann–Whitney U test for two groups and the Kruskal–Wallis test for more than two groups; when the overall Kruskal–Wallis test was significant, pairwise post hoc comparisons were performed using Bonferroni-corrected Mann–Whitney U tests. Categorical variables were compared using the χ^2^ test or Fisher’s exact test, as appropriate. The pre-PET versus post-PET distribution of the four surgical plan categories was tested for marginal homogeneity with the Stuart–Maxwell test.

To identify predictors of decision change after 18F-FDG PET/CT-informed MOOB reassessment, univariable binary logistic regression was performed for age, SUVmax of the index lesion, anatomic zone, metastatic burden, and ECOG performance status. Variables with *p* < 0.10 in univariable analysis were entered into the primary multivariable model. Given its established clinical relevance, metastatic burden was additionally evaluated in a separate sensitivity multivariable model. A further logistic regression was performed for the composite outcome of de-escalated management (cancellation plus de-escalation), reflecting overall downgrading of the initial surgical plan. Because of the modest sample size and small subgroups, all regression analyses were interpreted as exploratory and hypothesis-generating; no internal validation or model updating procedure was performed. Model fit was assessed using McFadden’s pseudo R^2^, and overall model significance was confirmed with the likelihood ratio test. Effect estimates are reported as odds ratios (OR) with corresponding 95% confidence intervals (CIs); for multi-level categorical predictors, both overall *p*-values and level-specific ORs are reported. A two-sided *p* < 0.05 was considered statistically significant.

## 3. Results

**Figure 1:**
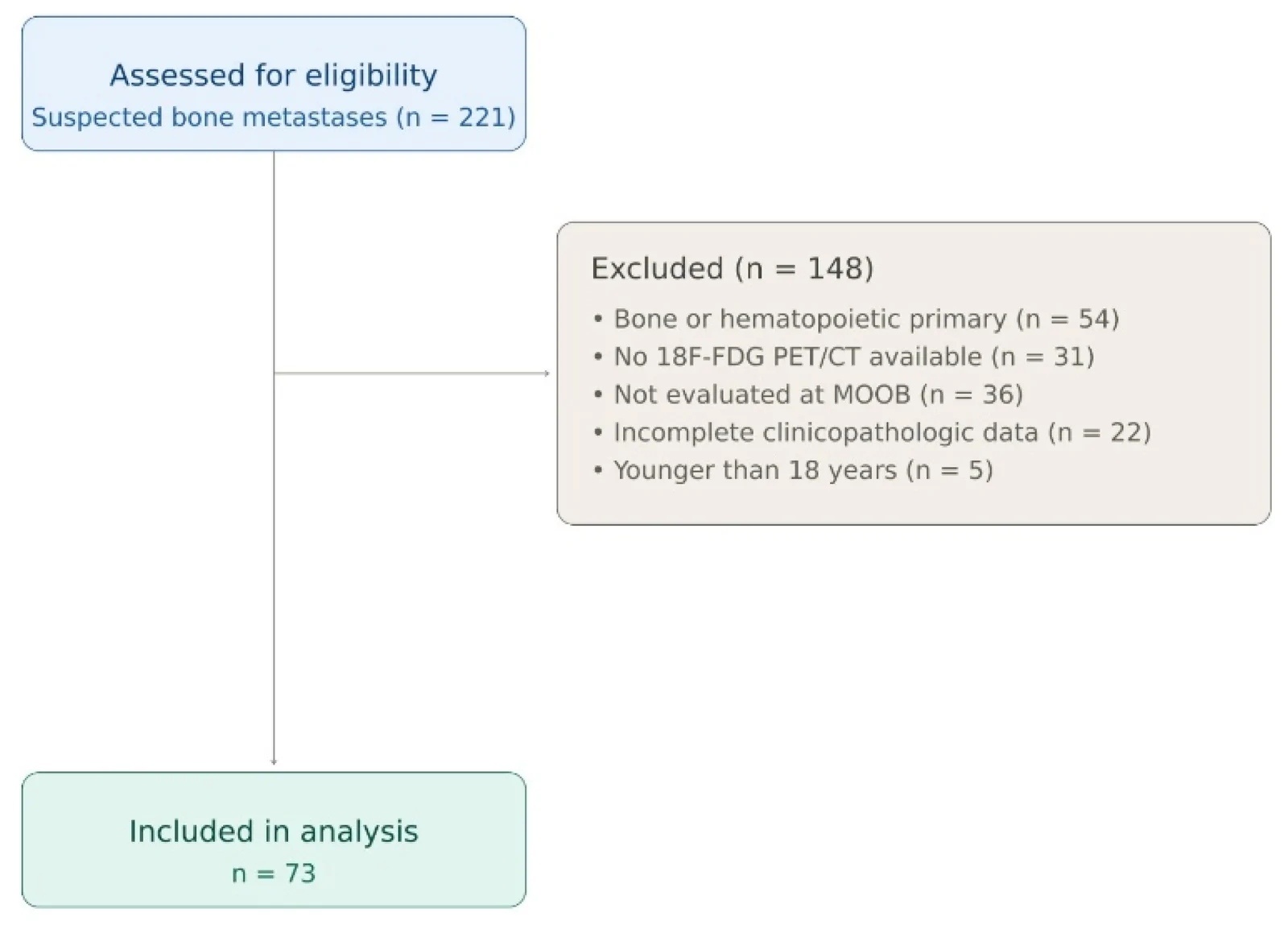
Flow diagram of patient selection (MOOB: Multidisciplinary Orthopedic Oncology Board; 18F-FDG PET/CT: fluorine-18 fluorodeoxyglucose positron emission tomography/computed tomography).

After the inclusion and exclusion criteria were applied, the study was conducted with 73 patients for analysis (Figure 1). The cohort had a mean age of 64.7 ± 12.1 years, with 39 (53.4%) males. The most frequent primary malignancies were lung carcinoma (n = 27, 37.0%), breast carcinoma (n = 16, 21.9%), and renal cell carcinoma (n = 10, 13.7%). The index metastatic lesion was in Zone 1 (axial skeleton/girdles) in 23 patients (31.5%), Zone 2 (long-bone epiphysis) in 27 (37.0%), and Zone 3 (long-bone diaphysis) in 23 (31.5%). Nine patients (12.3%) had solitary disease, 31 (42.5%) had oligometastatic disease, and 33 (45.2%) had polymetastatic disease. The median SUVmax of the index lesion was 10.4 (IQR 7.4 to 13.8), and 52 patients (71.2%) had an ECOG performance status of ≥2. Complete baseline characteristics are presented in Table 1. Because excluded cases lacked complete clinical, pathological, PET/CT, or MOOB decision data by definition, formal comparison of included and excluded patients was not feasible.

After 18F-FDG PET/CT review at the MOOB, the intended surgical plan was changed in 39 of 73 patients (53.4%) and maintained in 34 patients (46.6%). The distribution of the four surgical plan categories shifted significantly between the pre-PET and post-PET assessments (Stuart–Maxwell χ^2^ = 22.8, df = 3, *p* < 0.001) (Table 2). Two reciprocal shifts accounted for most of this redistribution: the proportion of patients directed to no surgery increased fourfold (6.8% to 28.8%), while palliative stabilization decreased correspondingly (69.9% to 47.9%). At the curative end of the spectrum, curative resection, absent from all pre-PET plans, was selected for 8 patients (10.9%) after PET/CT review, with a parallel decrease in palliative resection (23.3% to 12.4%).

When the same 39 plan changes were examined by direction of change, 20 (27.4% of the cohort; 51.3% of those changed) were surgery cancellations, 13 (17.8%; 33.3%) were escalations, and 6 (8.2%; 15.4%) were de-escalations. The primary reasons recorded in the MOOB minutes were redirection to radiotherapy (n = 11, 28.2%), estimated survival (n = 11, 28.2%), curative targeting (n = 9, 23.1%), ECOG status (n = 7, 17.9%), and detection of a new lesion (n = 1, 2.6%). Therefore, most decision changes reflected integrated PET/CT-informed multidisciplinary reassessment rather than the discovery of a new lesion alone.

The eight post-reassessment curative-intent recommendations are reported as intended treatment goals. The available MOOB minutes documented curative targeting as a primary reason for change in nine patients but did not consistently capture delivered surgery, margin status, or follow-up oncologic outcomes. Accordingly, these cases should be interpreted as curative-intent recommendations rather than confirmed curative treatments.

The SUVmax of the index lesion did not differ significantly between patients whose plan was changed and those whose plan was unchanged (median 11.2, IQR 7.5 to 15.6 vs. 9.6, IQR 6.9 to 13.1; *p* = 0.367), indicating that SUVmax was not a general predictor of any decision change. When patients were stratified by the direction of decision change, however, SUVmax differed significantly across the four management categories (Kruskal–Wallis H = 10.4, *p* = 0.015). In Bonferroni-corrected post hoc comparisons, canceled cases had a higher SUVmax than escalated cases (median 14.2, IQR 10.6 to 16.8 vs. 8.4, IQR 5.9 to 11.3; *p* = 0.031). In a complementary binary comparison, canceled cases also had a higher SUVmax than all non-canceled cases combined (median 14.2 vs. 9.5; *p* = 0.006).

Decision change varied significantly by anatomic zone of the index lesion (overall *p* = 0.004): the surgical plan was changed in 17 of 23 patients (73.9%) with a Zone 1 lesion, 16 of 27 (59.3%) with a Zone 2 lesion, and 6 of 23 (26.1%) with a Zone 3 lesion. In post hoc pairwise comparisons, Zone 1 lesions were associated with a higher decision-change rate than Zone 3 lesions (*p* = 0.010), whereas the differences between Zone 1 and Zone 2 (*p* = 1.000) and between Zone 2 and Zone 3 (*p* = 0.116) were not statistically significant.

Metastatic burden was not significantly associated with decision change overall (*p* = 0.066): the surgical plan was changed in 8 of 9 patients (88.9%) with solitary disease, 16 of 31 (51.6%) with oligometastatic disease, and 15 of 33 (45.5%) with polymetastatic disease. In a post hoc comparison of solitary versus non-solitary disease, solitary status was associated with an 8.5-fold increase in the odds of decision change (Fisher’s exact OR = 8.52, *p* = 0.032).

In univariable logistic regression, polymetastatic disease (vs solitary; OR 0.104, 95% CI 0.012 to 0.930; *p* = 0.043) and Zone 3 location (vs Zone 1; OR 0.125, 95% CI 0.033 to 0.465; *p* = 0.002) were associated with a lower likelihood of decision change, while higher ECOG performance score showed a borderline association (OR 1.550 per point, 95% CI 0.976 to 2.461; *p* = 0.063). Neither age (OR 1.010 per year; *p* = 0.601) nor SUVmax (OR 1.061 per unit; *p* = 0.281) was associated with decision change in univariable analysis.

In the primary multivariable model (without metastatic burden), Zone 3 location remained an independent inverse predictor of decision change (OR 0.125, 95% CI 0.033 to 0.483; *p* = 0.003), while ECOG performance score showed only a non-significant trend (OR 1.530 per point; *p* = 0.100; pseudo-R^2^ = 0.239). When metastatic burden was added in the prespecified sensitivity model, all three variables reached statistical significance: polymetastatic disease (vs solitary; OR 0.051, 95% CI 0.005 to 0.547; *p* = 0.014), Zone 3 location (OR 0.133, 95% CI 0.031 to 0.565; *p* = 0.006), and higher ECOG performance score (OR 2.109 per point, 95% CI 1.139 to 3.903; *p* = 0.018) emerged as independent predictors of decision change. The sensitivity model showed improved fit (pseudo-R^2^ = 0.363). Univariable and multivariable estimates are summarized in Table 3.

A prespecified secondary analysis evaluated predictors of de-escalated management, defined as the composite of surgery cancellation and de-escalation (n = 26 of 73). In this analysis, SUVmax of the index lesion was associated with downgrading of the surgical plan both in univariable (OR 1.189 per unit, 95% CI 1.052 to 1.343; *p* = 0.006) and in multivariable analysis (OR 1.241, 95% CI 1.052 to 1.464; *p* = 0.010). Zone 3 location remained an independent inverse predictor (OR 0.051, 95% CI 0.008 to 0.333; *p* = 0.002), and ECOG performance score showed a borderline association in the multivariable model (OR 1.823 per point, 95% CI 0.993 to 3.348; *p* = 0.053; pseudo-R^2^ = 0.297). Detailed univariable and multivariable estimates are presented in Table 4.

## 4. Discussion

In this single-center retrospective cohort with histopathologically confirmed bone metastases, PET/CT-informed MOOB reassessment was associated with a change in intended surgical plan in more than half of cases, with a statistically significant redistribution of the four surgical plan categories. Two reciprocal shifts dominated this redistribution: patients directed to no surgery increased fourfold (6.8% to 28.8%), while curative resection, absent from all pre-PET assessments, emerged as the post-PET option for 10.9% of patients. Polymetastatic disease, long-bone diaphyseal (Zone 3) location, and higher ECOG performance score were identified as independent predictors of a change in decision. SUVmax of the index lesion was not associated with overall decision change but was an independent predictor of de-escalated management, identifying patients in whom 18F-FDG PET/CT supported either cancellation or reduction in the planned procedure.

The 53.4% decision-change rate observed in our cohort exceeds the 20% to 45% range previously reported for the impact of 18F-FDG PET/CT on oncologic treatment strategies across diverse cancer populations [16,17,18]. In a registry analysis of patients with cancer of unknown primary, for example, Reinert et al. reported that PET/CT revised the treatment plan in 45.8% of cases and substantively altered the treatment goal in 26.5% [7]. Several factors may account for the higher rate observed in our study. First, our analysis was restricted to surgical decision-making within a dedicated MOOB framework, in which 18F-FDG PET/CT findings were systematically integrated alongside conventional imaging, ECOG status, and broader oncologic context [5,17]. Second, our cohort comprised exclusively patients with histopathologically confirmed bone metastasis, in whom the decision between aggressive local control and conservative management is particularly sensitive to additional information on disease extent and biological activity. The observed change therefore cannot be attributed to PET/CT alone; it reflects PET/CT-informed multidisciplinary reassessment in which imaging data were interpreted alongside medical oncology, radiation oncology, radiology, estimated survival, ECOG status, and conventional imaging findings. This attribution is clinically relevant because the MOOB process is the real-world mechanism through which PET/CT findings are translated into surgical recommendations.

Among the exploratory predictors identified in the multivariable model, the anatomical location of the index lesion exerted the strongest influence on decision change. Zone 1 lesions, comprising the axial skeleton and pelvic and shoulder girdles, had the highest rate of plan revision, whereas Zone 3 long-bone diaphyseal lesions had the lowest. This pattern is consistent with the more complex anatomic and biomechanical considerations that govern surgical decisions in the axial skeleton and with a growing trend toward non-operative management of selected spinal and pelvic metastases, facilitated by advances in systemic and radiation therapy [19]. The independent association of higher ECOG performance score with decision change similarly reflects the well-established role of patient performance status in metastatic disease management, where overall health status competes directly with technical surgical feasibility in determining treatment choice [3,20,21,22]. Importantly, ECOG was also recorded as a primary reason for plan change in some MOOB minutes, and this partial overlap should be interpreted as clinical reinforcement rather than as fully independent evidence.

A particularly informative aspect of our findings concerns the SUVmax of the index lesion. Although SUVmax did not differ between patients whose plan was changed and those whose plan was not, it was significantly higher in canceled cases than in escalated cases (median 14.2 vs. 8.4) and, in the prespecified secondary analysis, was independently associated with de-escalated management (OR 1.241 per unit). This pattern suggests that the influence of metabolic activity on surgical decision-making is directional rather than binary in this cohort. We deliberately avoid interpreting high SUVmax as a universal reason to avoid local intervention. SUV uptake varies by primary tumor histology, lesion size, lytic or blastic features, prior systemic or radiation therapy, scanner protocol, and baseline glucose level. This observation aligns with the broader literature, in which baseline SUVmax has been associated with prognosis and treatment response across multiple oncologic settings [23,24,25,26], and supports integrating metabolic data into surgical planning rather than restricting their use to diagnostic or follow-up purposes [21,24,26,27,28]. Thus, SUVmax should be regarded as a contextual variable that may contribute to cancellation or de-escalation when considered with systemic burden, expected survival, symptoms, and mechanical risk, rather than as a stand-alone marker of aggressive biology.

In practical terms, the patients most likely to benefit from PET/CT-informed MOOB re-evaluation may include those with suspected solitary or oligometastatic disease in whom curative-intent resection is being considered, patients with discordance between symptoms and conventional imaging, patients referred with incomplete systemic staging, and patients in whom occult systemic spread or poor performance status would meaningfully alter the extent of surgery. Conversely, in clearly polymetastatic patients with straightforward long-bone diaphyseal lesions requiring mechanical stabilization, PET/CT may be less likely to change the surgical category, although it may still be useful for systemic oncologic management.

The strengths of this study include the use of prospectively documented MOOB decisions at two predefined time points, a structured anatomic classification of the index lesion, and the application of complementary statistical approaches comprising the Stuart–Maxwell test of marginal homogeneity, multivariable logistic regression with a prespecified sensitivity analysis, and a separate composite analysis for de-escalated management. Several limitations should nonetheless be acknowledged. First, the design compares an initial orthopedic-team plan with a final PET/CT-informed MOOB recommendation; therefore, the study cannot disentangle the isolated effect of PET/CT from the broader effect of multidisciplinary reassessment. Second, the study measures changes in decision-making intent rather than verified treatment delivery. Actual surgery, margins, complications, post-cancellation pathological fractures, pain control, local progression, survival, functional outcome, and patient-reported outcomes were not systematically captured. Consequently, terms such as cancellation, de-escalation, and curative-intent recommendation should not be interpreted as proof that procedures were futile or that cure was achieved. Third, the single-center retrospective design limits generalizability, and the final cohort represented 73 of 221 screened patients; because excluded patients lacked key variables by definition, formal included-versus-excluded comparison was not feasible. Fourth, conventional imaging before the initial orthopedic plan was not standardized across all patients, and incomplete pre-PET staging may have amplified the apparent post-reassessment change. Fifth, the modest sample size, particularly in the solitary disease subgroup (n = 9), is reflected in wide confidence intervals, and the regression analyses should be viewed as exploratory and hypothesis-generating rather than definitive. Heterogeneity of primary tumors precluded tumor-specific subgroup analysis and may have introduced unmeasured confounding by tumor biology. The three-zone anatomic classification used in this study was developed to capture differences in surgical complexity and has not been externally validated; the magnitude of effect attributable to each zone may therefore differ in cohorts that employ alternative anatomic schemes. ECOG performance status was both abstracted as a documented reason for plan change in the MOOB minutes and tested as a numerical predictor in regression analysis; these two roles share information. Estimated survival was abstracted as a qualitative clinical estimate rather than a structured quantitative variable, reflecting real-world MOOB practice but precluding its formal incorporation as a regression covariate. SUVmax values were extracted from official nuclear medicine reports rather than re-measured under a standardized research protocol, and may reflect inter-reader and protocol variability inherent to clinical practice. Finally, patient-level transition-matrix data were not reliably retained in the de-identified analysis file; therefore, the study reports aggregate redistribution and directional categories rather than a full patient-level transition matrix.

## 5. Conclusions

18F-FDG PET/CT meaningfully reshaped surgical planning for more than half of patients with metastatic bone disease in our MOOB practice, both averting futile interventions and identifying previously unrecognized curative opportunities. The independent predictors identified here, namely polymetastatic disease, long-bone diaphyseal location, ECOG performance status, and SUVmax for the directional question, provide an evidence-based framework for prioritizing PET/CT in patients most likely to benefit from board re-evaluation. Prospective, multicenter studies are now needed to validate these findings, to refine criteria for selective PET/CT deployment, and to link PET-guided MOOB planning to downstream oncologic and functional outcomes, including local control, survival, and patient-reported function.

## Figures and Tables

**Table 1:** Baseline demographic and clinical characteristics (n = 73).

Characteristic	Value
Total patients, n	73
Age, years, mean ± SD	64.7 ± 12.1
**Gender, n (%)**	
Male	39 (53.4)
Female	34 (46.6)
**Primary tumor, n (%)**	
Lung	27 (37.0)
Breast	16 (21.9)
Renal cell	10 (13.7)
Urothelial	5 (6.8)
Prostate	4 (5.5)
Endometrial	4 (5.5)
Laryngeal	3 (4.1)
Pancreatic	2 (2.7)
Thyroid	1 (1.4)
Hepatocellular	1 (1.4)
**Anatomic zone, n (%)**	
Zone 1 (axial skeleton/girdles)	23 (31.5)
Zone 2 (long-bone epiphysis)	27 (37.0)
Zone 3 (long-bone diaphysis)	23 (31.5)
**Metastatic burden, n (%)**	
Solitary	9 (12.3)
Oligometastatic	31 (42.5)
Polymetastatic	33 (45.2)
SUVmax of index lesion, median (IQR)	10.4 (7.4–13.8)
**ECOG performance status, n (%)**	
0	2 (2.7)
1	19 (26.0)
2	25 (34.2)
3	17 (23.3)
4	10 (13.7)

Data are presented as n (%), mean ± SD, or median (IQR). SUVmax, maximum standardized uptake value; IQR, interquartile range; ECOG, Eastern Cooperative Oncology Group.

**Table 2:** Pre-PET/CT versus post-PET/CT surgical plan and test of marginal homogeneity.

Surgical Plan Category	Pre-PET, n (%)	Post-PET, n (%)	Difference	Direction
No surgery	5 (6.8)	21 (28.8)	+16	↑
Palliative stabilization	51 (69.9)	35 (47.9)	−16	↓
Curative resection	0 (0)	8 (10.9)	+8	↑
Palliative resection	17 (23.3)	9 (12.4)	−8	↓

↑: Increase or Approve of the Decision, ↓: Decrease or Decline of the Decision. Stuart–Maxwell test for marginal homogeneity: χ^2^ = 22.8, df = 3, *p* < 0.001. The table shows the aggregate redistribution of intended treatment categories after PET/CT-informed MOOB reassessment. The most notable shifts were a fourfold increase in patients directed to no surgery (6.8% to 28.8%) and the emergence of curative resection as a post-PET category (0% to 10.9%). These findings describe changes in decision-making intent and do not establish clinical futility, realized cure, or treatment superiority.

**Table 3:** Univariable and multivariable logistic regression for predictors of surgical decision change.

Variable	Univariable OR (95% CI)	*p*	Multivariable OR (95% CI), Without Burden	*p*	Multivariable OR (95% CI), with Burden	*p*
Age (per 1-year)	1.010 (0.972–1.050)	0.601	-	-	-	-
SUVmax (per 1-unit)	1.061 (0.953–1.181)	0.281	-	-	-	-
ECOG (per 1-point)	1.550 (0.976–2.461)	**0.063**	1.530 (0.922–2.538)	0.100	2.109 (1.139–3.903)	**0.018**
**Anatomic zone (overall)**	-	0.006	-	0.009	-	0.024
Zone 2 vs. Zone 1	0.513 (0.154–1.715)	0.279	0.482 (0.139–1.665)	0.248	0.362 (0.091–1.443)	0.150
Zone 3 vs. Zone 1	0.125 (0.033–0.465)	0.002	0.125 (0.033–0.483)	0.003	0.133 (0.031–0.565)	0.006
**Metastatic burden (overall)**	-	0.128	-	-	-	**0.049**
Oligometastatic vs. solitary	0.133 (0.015–1.197)	0.072	-	-	0.084 (0.008–0.865)	0.037
Polymetastatic vs. solitary	0.104 (0.012–0.930)	0.043	-	-	0.051 (0.005–0.547)	0.014

Odds ratios (ORs) with 95% confidence intervals (CIs) are reported. Variables with *p* < 0.10 in univariable analysis were entered into the primary multivariable model (pseudo R^2^ = 0.239). A sensitivity model additionally included metastatic burden (pseudo R^2^ = 0.363). Zone 1 and solitary disease served as the reference categories. ECOG, Eastern Cooperative Oncology Group; SUVmax, maximum standardized uptake value.

**Table 4:** Univariable and multivariable logistic regression for predictors of de-escalated management (n = 26 of 73).

Variable	Univariable OR (95% CI)	*p*	Multivariable OR (95% CI)	*p*
Age (per 1-year)	1.012 (0.972–1.053)	0.576	-	-
SUVmax (per 1-unit)	1.189 (1.052–1.343)	0.006	1.241 (1.052–1.464)	0.010
ECOG (per 1-point)	2.295 (1.348–3.909)	0.002	1.823 (0.993–3.348)	0.053
**Anatomic zone (overall)**	-	0.006	-	0.009
Zone 2 vs. Zone 1	0.452 (0.145–1.409)	0.171	0.290 (0.072–1.166)	0.081
Zone 3 vs. Zone 1	0.115 (0.027–0.500)	0.004	0.051 (0.008–0.333)	0.002
Metastatic burden (Poly vs. S/O)	1.061 (0.406–2.777)	0.904	-	-

De-escalated management was defined as the composite of surgery cancellation and de-escalation. Variables with *p* < 0.10 in univariable analysis (SUVmax, ECOG, anatomic zone) were entered into the multivariable model. Pseudo R^2^ = 0.297; likelihood ratio test *p* < 0.001. Zone 1 served as the reference category. OR, odds ratio; CI, confidence interval; ECOG, Eastern Cooperative Oncology Group; SUVmax, maximum standardized uptake value; S/O, solitary plus oligometastatic disease.

## Data Availability

The original contributions presented in the study are included in the article; further inquiries can be directed to the corresponding author.
